# Clinical factors and diagnoses associated with inappropriate urine-culture ordering in primary care

**DOI:** 10.1017/ash.2023.208

**Published:** 2023-09-29

**Authors:** Marissa Valentine-King, Barbara Trautner, Roger Zoorob, Michael Hansen, Jennifer Matas, Robert Atmar, Larissa Grigoryan

## Abstract

**Background:** Inappropriate urine-culture ordering is associated with increased antibiotic prescribing in myriad care environments, including acute and long-term care. In primary care, where urinary tract infections (UTIs) are commonly encountered, the appropriateness of urine-culture ordering has not been well described. We examined the appropriateness of urine-culture ordering and factors associated with inappropriate urine-culture ordering in primary care. **Methods:** We conducted a secondary analysis of data from a previous prospective study that included patients aged ≥18 years presenting with provider-suspected UTI with an accompanying urine culture at 2 safety-net, primary-care clinics in Houston, Texas, between November 2018 and March 2020. Patients with complicated or uncomplicated UTI were included, but those with a urinary catheter and pregnant females were excluded. Urine cultures were considered appropriate if the patient had an evidence-based symptom of UTI (ie, dysuria, frequency, urgency, hematuria, fever, chills, costovertebral angle tenderness, suprapubic, pelvic, or flank pain, or nephrolithiasis) as a diagnostic code or listed in providers’ free-text documentation. Diagnostic codes for symptoms that were not evidence based were grouped into categories based on body system, visit type (eg, routine visit), or sign or symptom clusters. We evaluated the relationships among demographic and clinical factors, the clinic visited, and non–evidence-based diagnostic codes with inappropriately ordered cultures. **Results:** We examined 870 cultures from 807 patients. Overall, 61.5% of patients were Hispanic (61.5%) and 23% were African American or Black. Also, 70.6% were women; the mean age was 49.2 years (SD, 14.6); and the mean Elixhauser score was 1.9 (SD, 5.4). Among the 870 cultures, 210 (24%) were ordered inappropriately. Dysuria (n = 289), frequency (n = 129), and UTI or cystitis (n = 117) were the most common, evidence-based codes among appropriate cultures. In the adjusted model, the nonteaching clinic (aOR, 6.33) and diagnostic codes comprising the following categories were associated with inappropriate culturing: acute lower back pain (aOR, 5.42), cardiac-related visits (aOR, 2.41), urinary incontinence (aOR, 4.46), routine health visits (aOR, 3.66), urine characteristics (aOR, 14.32), voiding difficulties (aOR, 3.88), and well-woman visits with a gynecological exam or family planning aspect (aOR, 12.27) (all *P* < .05). **Conclusions:** This research highlights potential gaps or miscues in provider behavior related to urine culture ordering, and unveiled problematic culturing related to urine characteristics and to routine visits, especially of a gynecological nature. This information can be incorporated into diagnostic stewardship interventions to address misconceptions, and to further explore the reasoning or processes wherein urine cultures are ordered for routine visits.

**Financial support:** NIAID UM1AI104681

**Disclosure:** None

